# Developing skilled doctor–patient communication in the workplace: a qualitative study of the experiences of trainees and clinical supervisors

**DOI:** 10.1007/s10459-017-9765-2

**Published:** 2017-02-20

**Authors:** Esther Giroldi, Wemke Veldhuijzen, Kristel Geelen, Jean Muris, Frits Bareman, Herman Bueving, Trudy van der Weijden, Cees van der Vleuten

**Affiliations:** 1 Department of Family Medicine, School for Public Health and Primary Care (CAPHRI), Maastricht University, Maastricht, The Netherlands; 2 Department of Educational Development and Research, School of Health Professions Education (SHE), Maastricht University, Maastricht, The Netherlands; 3 Department of General Practice, Erasmus University Medical Centre, Rotterdam, The Netherlands

**Keywords:** Doctor–patient communication, Qualitative research, Post-graduate education, Workplace learning

## Abstract

To inform the development of recommendations to facilitate learning of skilled doctor–patient communication in the workplace, this qualitative study explores experiences of trainees and supervisors regarding how trainees learn communication and how supervisors support trainees’ learning in the workplace. We conducted a qualitative study in a general practice training setting, triangulating various sources of data to obtain a rich understanding of trainees and supervisors’ experiences: three focus group discussions, five discussions during training sessions and five individual interviews. Thematic network analysis was performed during an iterative process of data collection and analysis. We identified a communication learning cycle consisting of six phases: impactful experience, change in frame of reference, identification of communication strategies, experimentation with strategies, evaluation of strategies and incorporation into personal repertoire. Supervisors supported trainees throughout this process by creating challenges, confronting trainees with their behaviour and helping them reflect on its underlying mechanisms, exploring and demonstrating communication strategies, giving concrete practice assignments, creating safety, exploring the effect of strategies and facilitating repeated practice and reflection. Based on the experiences of trainees and supervisors, we conclude that skilled communication involves the development of a personal communication repertoire from which learners are able to apply strategies that fit the context and their personal style. After further validation of our findings, it may be recommended to give learners concrete examples, opportunities for repeated practise and reflection on personal frames of reference and the effect of strategies, as well as space for authenticity and flexibility. In the workplace, the clinical supervisor is able to facilitate all these essential conditions to support his/her trainee in becoming a skilled communicator.

## Introduction

Doctor–patient communication is a core medical competency involving complex behaviour (Bensing 2003; Epstein 2013; Henry et al. 2013). In Salmon and Young (2011) called for a shift from ‘communication skills’ towards ‘skilled communication’ in communication training and research. They argued that communicative behaviours are too complex to be predetermined and assessed with behavioural checklists. A holistic and context-specific approach would be more appropriate to address this complexity (Salmon and Young 2011). This view is empirically supported by studies that have demonstrated both the context-specific and goal-directed nature of communication in daily practice (Essers et al. 2011, 2013; Giroldi et al. 2014; Veldhuijzen 2011; Veldhuijzen et al. 2013), as well as the transfer gap in generic communication training (Kramer et al. 2004). As a consequence, it is believed that learning skilled communication cannot take place only in a teaching setting isolated from the clinical context, but is a continuous process in interaction with the clinical environment. Hence, there is growing attention for the role of the clinical workplace in learning communication (Salmon and Young 2011; van den Eertwegh et al. 2013, 2014).

Several empirical studies and theories have emphasised the relevance of certain elements to learning communication in the workplace. Given that learning communication involves a complex interplay with the social context, we adopt a social constructivist perspective (Mann 2011). Within social constructivism, and also within learning communication in particular, the learner’s attitude is viewed as an important element. A theory in which attitude has a key position, and which also has been suggested to be a relevant theory for future communication research, is Mezirow’s Transformative Learning Theory (Aper et al. 2015; van den Eertwegh et al. 2014). A key concept in this theory is the learner’s frame of reference, that is, one’s personal assumptions, knowledge, beliefs and emotions. According to Mezirow, an experience is the starting point for learning. Becoming aware of one’s own frame of reference and adjusting it accordingly through reflection on this initial experience creates scope for behavioural change (Mezirow 1997). At such point, however, the learner still needs to develop effective and useful behaviours that fit the new frame of reference. Taking again into account the social context, and as demonstrated in the literature, good clinical role models and practical rehearsal are vital to this process (Côté and Leclère 2000; Silverman 2011; Stok-Koch et al. 2007; van den Eertwegh et al. 2014). Mimetic Learning Theory, recently developed by Billet, describes how people learn in workplaces through a process called ‘mimesis’, in which the learner actively engages with others (e.g. observing behaviour), imitates and rehearses to accomplish the required performances (Billet 2014).

The clinical supervisor, from his position as a mentor, coach and role model, is both a powerful inspirer for engagement in mimetic learning, and an influential reference point with whom learners compare their frames of reference. Hence, to be able to understand how to facilitate the learning of doctor–patient communication in the workplace, it is important to understand in detail not only how learners learn communication, but also how clinical supervisors can make optimal use of their role to facilitate this learning. The main aim of this qualitative study therefore is to obtain insight into the experiences of trainees and supervisors regarding how trainees learn communication and which supervisor behaviours support this learning in the workplace. Understanding these experiences may serve as a starting point in the development of recommendations for supporting trainees’ learning.

## Methods

### Setting

This study was set in the context of General Practice (GP) specialty training in the Netherlands, which offers its trainees medical communication training during weekly peer group sessions at the training institute. These sessions are guided by two qualified trainers: a behavioural scientist and a GP. GP supervisors also receive communication training and didactic skills training to support trainees in learning communication. Training for supervisors is offered during monthly peer group sessions at the training institute and supervised by the same trainers as the trainee group.

### Ethics

The Netherlands Association for Medical Education (NVMO) granted its approval for our study protocol. Participating trainees and supervisors gave written informed consent. All data were anonymized with codes.

### General design

We used various sources of qualitative data (triangulation) to obtain a rich understanding of trainees’ and supervisors’ perspective on learning communication: focus group (FG) discussions, discussions during training sessions and individual interviews. Data were collected alternately from supervisors and trainees, allowing findings arising from one group to inform data collection in the other group. Guided by sensitizing concepts derived from existing relevant theoretical concepts (Bowen 2006), we applied constructivist grounded theory principles, i.e. iterative data collection and analysis, constant comparison and purposeful sampling (Watling and Lingard 2012), to develop a conceptual model on trainees’ learning. To identify common themes and their relationships, we performed a thematic network analysis (Attride-Stirling 2001).

### Data collection

#### Focus group discussions with supervisors

**Fig. 1:**
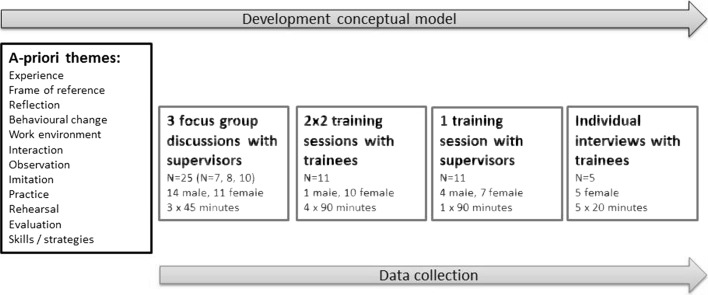
Process of data collection and analysis

We organised focus-group discussions with GP supervisors (Fig. 1) since we expected that an interactive setting would stimulate reflection on one’s own experiences and enable the expression of latent thoughts and beliefs. We invited 60 supervisors from two different GP specialty training centres, of which 25 supervisors participated. Their teaching experience as a supervisor ranged from 1 to 25 years (mean: 6.7 years). Three focus-group discussions were organised at the training institute. An experienced chair (JM/WV/FB) guided the discussions and the primary researcher (EG) was present to take minutes. In order to stay close to daily practice, we jump-started the discussions by presenting communication issues GP trainees faced in real practice, such as communicating with talkative patients. Supervisors were invited to reflect on how trainees overcome these challenges in practice and how they support their trainees in this process.

#### Training sessions with trainees

In order to follow closely the actual learning process of trainees, we used communication training sessions at the GP training centre as stimuli for discussion (Fig. 1). We asked two existing first year communication training groups to participate. Each group participated in two group sessions guided by EG or WV. During the first session, trainees discussed a new communication topic, formulated learning goals related to this topic and extensively discussed how they planned to work in practice on their learning goals. Throughout the second session, which took place two weeks later, trainees discussed what they had done in practice and what had helped them to achieve their learning goals.

#### Training session with supervisors

Similar to the aforementioned training sessions with trainees, we used a training session on communication and didactics for supervisors at the institute as a stimulus to generate relevant data (Fig. 1). We invited an existing training group of 11 supervisors to participate in the study. These were different supervisors than the supervisors participating in the focus group discussions. Their teaching experience as a supervisor ranged from 2 to 5 years (mean: 2.6 years). The session started with a 30-min plenary discussion on how supervisors planned to support their trainees in tackling communication issues. Subsequently, supervisors were divided into four small groups, each led by a facilitator. During these 60-min sessions, GP supervisors practised learning dialogues with their trainees through role play, after which the facilitator probed supervisors about what they had done during this dialogue and why.

#### Individual interviews with trainees

To obtain a more in-depth understanding of some specific elements of trainees’ learning process, we invited the 11 trainees from the focus groups to participate in an individual interview 2–3 months after the second training session (see ‘Training sessions with trainees’). Five trainees were interviewed by EG, who probed them about how they had worked on their learning goals in clinical practice during the past months.

### Data analysis

**Fig. 2:**
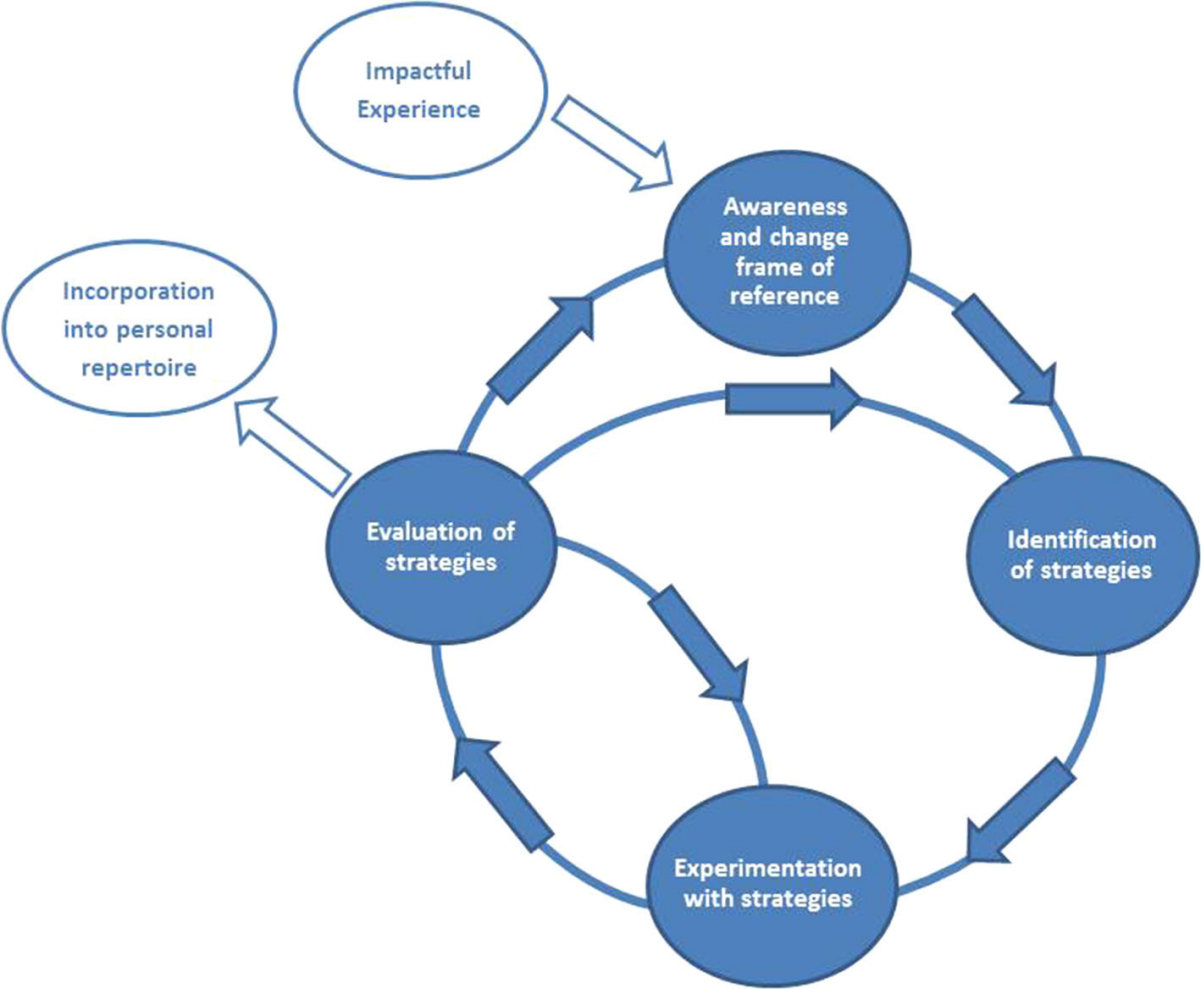
Trainees’ communication learning process

All discussions and interviews were audiotaped and transcribed verbatim. At least two independent researchers with different backgrounds (EG: Health Sciences; KG/WV: Medicine) coded the transcripts using specialised software (ATLAS.ti). First, we derived a priori themes from Transformative Learning Theory and Mimetic Learning Theory (Fig. 1). During the analysis of the first focus group with supervisors, we identified fragments relevant to how trainees learn and how supervisors support this learning. Fragments that fit the a priori themes were coded as such, while we also defined new themes. Themes were organised into a thematic network to visualise relationships (i.e. between main themes and between main themes and subthemes). The coding structure that ensued guided the analysis of all subsequent (focus-group) discussions and individual interviews. During this process, we constantly grouped, split and refined themes and modified the thematic network. The network and written memos with interpretative and reflexive thoughts and ideas about the data informed the development of a conceptual model visualising trainees’ learning process (Fig. 2). The analysts discussed differences in coding until consensus was reached. The thematic network and conceptual model were repeatedly discussed with all co-authors.

### Data saturation

The main themes that eventually resulted in the conceptual model were present in every dataset. In the first dataset (i.e. focus group discussions), we identified subthemes related to all of the final main themes. The analysis of the second and third dataset (i.e. training sessions) led to the identification and a deeper understanding of the main themes and sub themes. The fourth dataset (i.e. individual interviews) clarified the ways in which the phases of the learning process took place in practice, such as the evaluation of strategies. After analysing the different sources of qualitative data, we felt that we had obtained a rich understanding of trainees’ learning process and supporting factors, and data saturation was considered to be reached.

## Results

### Trainees’ learning process

We were able to organize trainees’ and supervisors’ reflections about trainees’ learning into six phases that together constitute the process of how trainees learn effective doctor–patient communication (Fig. 2).

#### Phase 1: impactful experience

Trainees and supervisors described that the learning process of a trainee is usually set in motion by an impactful experience, such as a challenging patient encounter or feedback from a peer, supervisor or trainer. Such an experience triggers trainees’ intrinsic motivation to learn to handle similar situations more effectively and typically forms the basis for reflection.

Well yes, I am aware of certain deficiencies, which is why I am eager to learn. I notice that when I stumble, I start to practice these things.

Individual interview with trainee

#### Phase 2: awareness and change frame of reference

Upon reflection with others, trainees’ initial feeling about the impactful experience - ‘this encounter did not feel quite right’, for example—becomes more explicit. This process helps trainees to become aware of their actual behaviour and of the feelings, knowledge and attitudes that may have given rise to the behaviour of that moment. Awareness and reflection create opportunities for changes in their frames of reference, for instance in their feelings and attitudes towards patients or assumptions about what makes good communication. As a result, trainees often wish to change their behaviour, by bringing their communication style in line with the new beliefs and attitudes.

It all depends on how the trainee experiences a consultation; is he uncomfortable? Does he notice that in time? Does he act on it? What is often the case in the beginning is that it just happens to them, so to speak. The next thing you get when you discuss it with them is the phase in which they become aware of it, they wake up as it were, and they try to regain control by changing the way they communicate. So the moment they themselves sense it, they become more watchful.

FG with supervisors

#### Phase 3: identification of strategies

When new effective behaviours are not yet available, trainees try to identify possible strategies through observations of and discussions with their supervisor, by discussing alternatives with their peers during communication training or by reading the communication literature. Any specific wording, few words, or sentence that they can find may be helpful.

I am often searching for the right words, so yes, sometimes it is nice to just, yes, very childish, anyway to just have a clear sentence like: well yes, throw it in, right? And see what happens.

Individual interview with trainee

#### Phase 4: experimentation with strategies

After identifying alternative strategies, trainees then try them in practice. The decision to do so, however, must be a deliberate one, as trying out new behaviour takes up a lot of cognitive space and it is easy to revert to usual routine reactions. Trainees therefore use memory aids such as sticky notes to remind them to try out a new strategy during patient encounters. During this experimentation phase, trainees pay attention to wording and timing, as well as to the appropriateness of strategies in light of the situation.

If I do not consciously think of it beforehand, then I will probably forget it (…). You must really make yourself aware of it in advance, like: OK, during this encounter I will make sure that somebody is being put at ease. That way you really focus on the reassurance, and then it does work.

Individual interview with trainee

#### Phase 5: evaluation of strategies

After trying out a strategy, trainees evaluate its effectiveness by asking themselves questions such as: (1) Does it reflect my personal style?; (2) Is it effective, i.e., have I achieved my consultation goal?; (3) Do I apply it correctly?; and (4) Does it fit the situation at hand? When applying the strategy, trainees use their feelings and patient’s responses as outcome measures. This fifth phase eventually results in one of three different outcomes: (1) trainees consider the strategy ineffective and immediately search for alternatives (Fig. 2, middle loop); (2) they consider the strategy effective and continue to practise to achieve refinement, e.g. by reformulating it in such a way that they feel more comfortable with it.(Figure 2, inner loop); and (3) the evaluation changes their frame of reference (Fig. 2, outer loop), for instance when trainees realise that respectfully interrupting a talkative patient does not harm the doctor–patient relationship, thereby changing their personal feelings and assumptions about interruptions.

So, when you ask that question, at some point you notice that: this is up my street, this is not up my street, this works, or this does not work. And to use that same sentence or strategy again in another situation. And when you realise that: well, this still does not work, then you put it aside and you think: yes, this is not up my street or I do not notice any desired effect.

Individual interview with trainee

Well, if trainees perceive that something works just fine and that it is OK to do so, then the next time they do it more easily.

FG with supervisors

#### Phase 6: incorporation into personal repertoire

Through repetition and rehearsal, the new communication strategy, rather than being a technique learned from others, gradually becomes part of the trainees’ personal repertoire. The learner has now reached a point where the respective strategy is applied automatically, not deliberately. In this final phase, trainees are able to apply the strategies in their repertoire adaptively to the particular demands of the context.

In the beginning you try to memorise standard sentences. And now you do it your own way and then it will come to you, without having to constantly think: request for help, request for help, request for help. Because you start practising, you are really using it, and when I am using it, it sticks with me, yes, in a way that I feel is right, say, like, and reflects my personal style, so now it is a part of me.

Individual interview with trainee

When you face something for the first time, you have no clue what to do. And what you see at some point is that trainees do recognise such a situation, but they know just one way to solve it. By practising and seeing it often, you also learn other strategies and eventually you know: this strategy might just work in this particular situation.

FG with supervisors

### Supervisors’ actions to support trainees’ learning

**Fig. 3:**
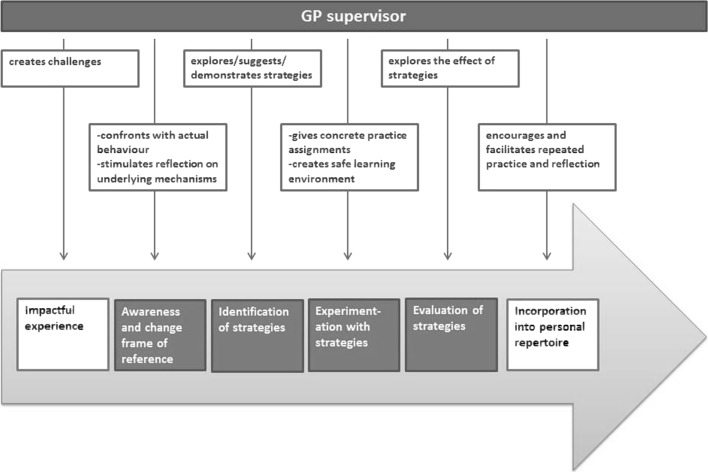
Supervisors’ actions to support trainees in learning communication

For every phase of a trainees’ learning process, supervisors and trainees described actions that supervisors perform to support trainees’ learning. Supervisors’ actions in each specific phase are presented in Fig. 3 below. It must be noted, however, that this separation is somewhat artificial: in reality, the process is more integrated as actions flow naturally from one into another and can be applied within a very short time span.

#### Impactful experience

Most of the time, supervisors let their trainees decide which experiences will be brought up for discussion. Occasionally, however, they deliberately place their trainees in challenging situations by scheduling specific consultations in their trainees’ diary. These are consultations of which supervisors suspect that they require communication competencies that their trainees have not yet mastered or actively worked on.

I think that the trainee must first have experienced it himself. So the first thing I do is to assign such a patient to the trainee and then we will see. You must have experienced it first before you know what to do with it.

FG with supervisors

#### Awareness of and change in frame of reference

To make trainees mindful of their frame of reference, supervisors confront them with their communication behaviour. They do so by viewing video-recorded consultations together with their trainees and discussing relevant instances, e.g. in case of prominent non-verbal cues or when the trainee feels he/she could have done something differently. They then ask their trainees to reflect upon the observed behaviour and the underlying thoughts, feelings and assumptions that triggered it.

And he keeps on pressing the “pause” button to ask: ‘What happened there?’; ‘What do you notice that the patient does?’ ‘Have you noticed it as well?’. Then I realise that, many things do go unnoticed. Really, it’s only when you see the video played back. Suddenly you get it, like: ah, yes, that is the reason or this is what you feel or this is what is happening.

Individual interview with trainee

While a video-recorded consultation is the most common starting point for raising trainees’ awareness of their frame of reference, some supervisors use an observed consultation (i.e. supervisor observes trainee) or a duo-consultation (supervisor and trainee see the patient together) for this purpose.

We are talking about patients, but also about the fear, uncertainty and concerns that may be felt by the trainee, right: ‘Did I do the right thing?’ I often spell it out, you know. ‘Were you afraid of that?’ Yes, yes, the unknown, that uncertainty indeed.

Training session with supervisors

Once this frame of reference has been made explicit, they challenge their trainees to look at the encounter from a different perspective by helping them understand the underlying mechanisms of the interaction: Why did the patient and trainee communicate or respond the way they did? To promote understanding, supervisors focus on trainees’ feelings of uneasiness and how these relate to their behaviour. In addition, they seek to arouse the trainees’ curiosity about the patient’s background. Both strategies aim to promote changes in how trainees interpret the patient’s communication. Promoting insight into the patient’s reactions helps trainees to view the encounter or the patient as a challenge instead of a burden.

I say: ‘what happened to you there?’. ‘Hmm, yes. I got rather annoyed’.’OK, so what did you do with that feeling?’.’Well, nothing, really’.’You get annoyed but you just lean back calmly in your chair. That’s not right, that’s difficult for the patient.’

FG with supervisors

If you can counterbalance it and say: ‘That may be so, but perhaps he has a reason for that?’ That way you arouse their curiosity.

FG with supervisors

Furthermore, supervisors challenge trainees’ existing assumptions about what ‘good communication’ entails. They try to make clear that, although skills are important, trainees also need to learn occasionally to ‘let go’ of the general skills and structure they have learned during their communication training. It is important to be flexible and accept that it is not always possible or even desirable to apply all skills in one consultation; sometimes they may be counterproductive and should therefore be avoided or adjusted to the context. Finally, supervisors stress that there is a need for authenticity and individuality: trainees must be free to use the communication strategies that are appropriate to the clinical context as well as to their personal style.

Do something you did not learn to do with the communication technique you acquired at the institute. Trying to apply everything you learned will not work for every patient. Patients that should be cut short require a different approach. Patients of a different origin require yet another approach. So it is something you should learn: it cannot always be done the same way.

FG with supervisors

#### Identification of strategies

Since trainees often struggle to find the right words, they appreciate it when supervisors suggest concrete words or sentences that they can try out. Instead of suggesting sentences, some supervisors stimulate trainees to develop their own formulations to ensure a closer alignment with the trainees’ personal communication style.

What I do is not always what is best for the trainee. This is why I let them think through for themselves over ways to regain control. As far as developing your own, individual style goes, that’s different for each trainee. They have to discover for themselves how they can best do it.

FG with supervisors

Supervisors also invite trainees to observe them during consultations, while repeating that the style of the trainee need not necessarily reflect their own. Exact copying of observed behaviour may have unexpected effects due to differences in gender, age, position and background between trainee and supervisor. Nonetheless, such observations afford trainees the opportunity to become acquainted with alternative behaviours and to witness their outcomes in a variety of settings. Trainees can then adjust observed strategies in such a way that they feel comfortable using them, while still achieving the desired outcome.

You can carry on talking about it but, at a certain point, the chat has to turn into concrete action . This is why I say you should use every possible opportunity for observation: at the GP out of hours service, during visits and consultation hours. Use all these and then you see the greatest possible variety. Right, and that is exactly our skill, to know when to adapt, various models. He should also have the opportunity to observe you in all kinds of situations.

FG with supervisors

#### Experimentation with strategies

Given that trying out new behaviours does not tend to be an automatic process for trainees, supervisors actively encourage trainees to practice. For instance, they instruct trainees to try out a specific sentence during a patient encounter, and sometimes first let them rehearse that strategy in a safe environment by means of a role-playing exercise. They also have trainees follow up challenging patients to ensure continuity, which fosters a trusting doctor–patient relationship, and, in turn, facilitates effective communication. At the same time, continuity allows trainees to try out different strategies on the same patient and to evaluate their effects, helping them discover what works best in the respective situation.

To maximise the benefit of this experimentation phase, a safe learning environment was viewed as vital: developing a personal style becomes easier when the trainee feels comfortable experimenting with different behaviours. Therefore, supervisors reassure trainees that it is okay to test alternatives and that, should they fail, they would still be able to find a solution together. Also by creating openness, such as admitting that they too find certain situations challenging, they put trainees at ease.

I think it is also important to reassure the trainee by saying that it is okay to make a mistake during an encounter.

FG with supervisors

A final aspect related to safety is trainees’ fear of making medical errors. Diagnostic uncertainty hampers trainees in improving their communication. Hence, it is important that trainees gain confidence in their medical knowledge, so that more cognitive space becomes available for the trainees to focus on communication issues.

Another important learning goal is getting some grip on the consultation, feeling that you can manage an entire consultation from beginning to end. Trainees often feel that they lack a sense of alarm. Especially in the beginning of training, they are afraid to make a mistake. In that case you could focus first on discussing alarming signals with them.

FG with supervisors

#### Evaluation of strategies

To evaluate the effectiveness of the applied strategies, supervisors ask probing questions such as: ‘How did you notice that your strategy was or was not effective?’, ‘Which verbal or non-verbal cues did the patient give?’ and ‘How did you verify whether your interpretation of the effect is correct?’. During this discussion, they also sometimes revert to the trainees’ frame of reference by exploring how they felt when employing the strategies and whether this experience changed their attitudes towards patients or the way they see their communication habits. By making trainees aware of the change in their frame of reference, supervisors try to consolidate this change.

You can also start playing around together along the lines of,’What do you do there?’ and ‘How did that work?’ and ‘Why do you think it worked better this time than last?’. Because you are talking about it, they can deliberately try things and then see how they work out and if they work. In this way, they also learn about what approach to use; what they feel comfortable using; what works with this particular patient? Because, after all, every case is, in itself, unique.

FG with supervisors

#### Incorporation into personal repertoire

In order for trainees to integrate new strategies into their personal repertoire, it was considered essential that trainees be encouraged to focus on one communication theme at a time so as to avoid cognitive overload. They facilitated this process by encouraging repeated practice and reflection, for instance through repeated administration of concrete practice assignments, or by encouraging trainees to follow up patients and scheduling follow-up reflection sessions to discuss the effect of strategies.

Then you discuss it and suddenly another door opens and the next time she takes a different course and then you look at her, like: ‘Did it work for you?’ And then you bring it up again and again and just follow up such a patient over time in the next months.

FG with supervisors

That is really just a small issue, which is really very specific, and that is what continues to be your focus until you notice that it has been absorbed. After that, there will be something else, of course.

Training session with supervisors

## Discussion

In this study we developed a conceptual model of how trainees learn to become skilled communicators and how supervisors support this process in the workplace. Interaction with patients and supervisors causes trainees to reconsider their frame of reference, and, consequently, to revisit their communication behaviours. By going through a continuous learning cycle of identifying, testing and evaluating alternatives, trainees learn to develop a personal repertoire of effective communication strategies that reflects their frame of reference. In every phase of this learning cycle, GP supervisors take specific action to facilitate the process.

A recent study by van den Eertwegh et al. also explored the communication learning process of GP trainees (van den Eertwegh et al. 2015). The authors identified a five-phase learning model which bears much resemblance to our learning phases. Hence, the studies’ findings are mutually reinforcing: both stress the value of repeated practice and reflection to ensure that communication strategies are integrated into the learners’ personal repertoire. Similarly, both studies reiterate the importance of revisiting the learner’s frame of reference, as did our previous study which explored communication issues encountered by trainees (Giroldi et al. 2015). At the same time, we observe that our findings overlap considerably with generic models of reflective learning, such as the learning cycles of Korthagen et al. (2001). What this study adds, however, is the insight that learning communication is very contextualised and personalised. There is more to being a skilled communicator than the mere mastery of a set of predetermined skills (Salmon and Young 2005, 2011). It involves being able to recognise a certain context and adapt the use of communication strategies accordingly. To be able to do so, trainees need impactful experiences, critical reflection on personal feelings and assumptions and repeated practice and evaluation. The resulting communication repertoire is highly idiosyncratic, that is, shaped to the individual.

Our paper also extends existing learning models by reinterpreting the role the clinical supervisor plays in learning communication in the workplace. Unlike previous literature suggesting that clinical supervisors may be negative communication role models (Côté and Leclère 2000; Egnew and Wilson 2011; Haidet et al. 2002, 2005; Kramer et al. 2004; Vernooij-Dassen et al. 2000), the supervisors in this study, who have already received extensive training in communication skills and didactic skills, seem to offer valuable support in every step of the communication learning process. Trainees do not feel comfortable being left to their own devices when it comes to learning communication. Instead, they need concrete examples (e.g. words, sentences), a safe learning environment in which there is space for trying out alternatives and individuality, as well as repeated confrontation with and reflection on their own and the patient’s communication behaviour and its underlying mechanisms. The supervisor can facilitate and nurture all these essential conditions, allowing their trainee to develop into a skilled communicator. Our findings demonstrate how principles of Transformative Learning Theory (Mezirow 1997) and Mimetic Learning (Billet 2014) inform our understanding of how trainees potentially learn skilled communication. This study confirms the view that trainees learn through interaction with others (e.g. peer, trainer, and supervisor) and by continuously evaluating and adjusting behaviour and their underlying frames of reference.

### Strengths and limitations

The use of data, method *and* theory triangulation has enhanced the credibility of our findings (Frambach et al. 2013). Using the experiences of both trainees and supervisors has given us a rich understanding of how trainees learn medical communication in the workplace. Our strategy to combine individual interviews, focus groups and discussions during communication training sessions facilitated in-depth reflection as well as close proximity to actual practice. However, since we did not perform any naturalistic observations of what happens in the workplace, we cannot draw conclusions on whether our findings truly represent the actual learning practices. Moreover, we cannot draw conclusions on what ideally should happen to support trainees’ learning. Triangulating two theories with empirical data has allowed us to test and refine our understanding of these theories in the specific context of communication learning. By iteratively collecting and analysing the data until no new themes could be identified, we ensured that our findings were consistent with the GP context. Nonetheless, we should refrain from making any inferences regarding the generalisability of our findings to other medical specialties or the actual effectiveness of supervisor support on trainees’ learning outcomes.

### Recommendations for future research

Further validating our conceptual learning model by observing trainees in the clinical setting as they move from novices to experts, exploring how the model can be used for communication training in the clinical setting, and studying the actual effectiveness of supervisors’ support in facilitating trainees’ learning are three important areas for further research. To allow extrapolation of our findings, trainees’ communication learning process needs to be explored in other medical specialties as well. In addition, future studies could investigate whether our model is also applicable to clinical competencies other than medical communication that involve complex behaviours to be learned in the working environment, such as professionalism, shared decision-making and collaboration.

## Conclusions

In communication training, attention needs to be paid to how learning communication takes place in clinical practice. After further validation of our findings, we may conclude that the workplace should allow trainees to develop their personal communication repertoire, which may require being able to work on a communication theme for a longer period of time; space for reflection on behaviours and underlying frames of reference; an environment in which trainees feel safe to practise alternatives; and opportunities to evaluate the effect of strategies. The latter also necessitates trainees following up on their own patients. Considering the significance of the supervisor’s role, it is important supervisors receive didactic training in which they obtain insight into their trainees’ learning process and what they can do to support this.
